# Fibrillary Glomerulonephritis Diagnosis Is Enhanced by DNAJB9: Three Cases with Different Clinical, Anatomopathologic Features and Outcomes

**DOI:** 10.3390/pathophysiology32020022

**Published:** 2025-05-25

**Authors:** José C. De La Flor, Marco Dominguez Davalos, Tania Linares Grávalos, Marina Alonso-Riaño, Francisco Díaz, Celia Rodríguez Tudero, Rocío Zamora González-Mariño, Michael Cieza Terrones, Jesús Hernández Vaquero

**Affiliations:** 1 Department of Nephrology, Hospital Central Defense Gomez Ulla, 28047 Madrid, Spain; tlingra@mde.es (T.L.G.); jherva5@mde.es (J.H.V.); 2 Health Sciences Doctoral Program, Faculty of Medicine, Alcala University, 28805 Madrid, Spain; 3 Department of Medicine and Medical Specialties, Faculty of Medicine, Alcala University, 28805 Madrid, Spain; 4 Department of Nephrology, Hospital Cayetano Heredia, Lima 15002, Peru; marco.dominguez.d@upch.pe; 5 Faculty of Medicine, Peruana Cayetano Heredia University, Lima 15002, Peru; 6 Department of Anatomic Pathology, Hospital 12 de Octubre, 28041 Madrid, Spain; marina.alonso@salud.madrid.org; 7 Department of Anatomic Pathology, Hospital Gregorio Marañón, 28008 Madrid, Spain; fdiazc@salud.madrid.org; 8 Department of Nephrology, Hospital Universitario de Salamanca, 37007 Salamanca, Spain; crodrigueztudero@usal.es; 9 Surgery Doctoral Program, Faculty of Medicine, University of Salamanca, 37007 Salamanca, Spain; 10 Department of Nephrology, Hospital Universitario General Villalba, 28400 Madrid, Spain; rocio.zamora@hgvillalba.es; 11 Department of Engineering, Faculty of Science and Engineering, Peruana Cayetano Heredia University, Lima 15002, Peru; michael.cieza@upch.pe

**Keywords:** fibrillary glomerulonephritis, rapidly progressive glomerulonephritis, DNABJ9

## Abstract

**Background:** Fibrillary glomerulonephritis (FGN) is a rare and poorly understood kidney disease characterized by the deposition of non-amyloid fibrils in the glomeruli. Its clinical heterogeneity and high rate of progression to end-stage renal disease (ESRD) pose significant diagnostic and therapeutic challenges. This case series aims to enhance awareness of FGN and emphasizes the need for further research to improve patient outcomes. **Case Reports:** We reviewed the clinical, histopathological, and therapeutic data of three patients with FGN diagnosed by kidney biopsy. The cases included variations in clinical presentation from nephrotic syndrome to rapidly progressive glomerulonephritis (RPGN). Diagnostic methods incorporated light microscopy, immunofluorescence, and electron microscopy, with the integration of DnaJ homolog subfamily B member 9 (DNAJB9) staining for confirmation. Patient 1 showed a more favorable response to rituximab, achieving complete remission (CR) at 6 months and maintaining CR after 3 years. Patient 2 showed only partial remission after 2 years following treatment with rituximab. Patient 3 presented with RPGN and rapidly progressed to ESRD despite aggressive immunosuppressive therapy. **Discussion:** DNAJB9 has emerged as both a specific and sensitive biomarker in patients with FGN and has facilitated accurate differentiation from other glomerulopathies. This series underscores the variability in clinical outcomes and responses to therapy as well as the importance of early and accurate diagnosis. **Conclusions**: FGN remains a diagnostic and therapeutic challenge due to its rarity and heterogeneity. Advances in biomarkers like DNAJB9 have improved diagnostic accuracy, distinguishing FGN from similar conditions such as immunotactoid glomerulopathy. Further research into pathophysiological mechanisms and targeted therapies is essential to optimize management and outcomes for affected patients.

## 1. Introduction

Fibrillary glomerulonephritis (FGN) is an immune complex-mediated glomerulonephritis and a rare, poorly understood glomerular disease, accounting for less than 1% of glomerulopathies diagnosed by kidney biopsy (KB). Despite its low prevalence, FGN has a significant clinical impact due to its potential to cause irreversible kidney damage. This condition is characterized by the deposition of non-amyloid, Congo red-negative fibrils measuring 16 to 24 nm in diameter, as observed through electron microscopy (EM). Although first described in the 1980s, its pathophysiology and underlying immunological mechanisms remain an active area of research. While FGN is typically idiopathic, growing evidence suggests it may be an autoimmune disorder driven by complement activation, primarily via the alternative pathway, and by the deposition of immunoglobulins (Igs), predominantly of the IgG4 subtype [1]. Moreover, up to 50% of patients with FGN have a history of underlying conditions, including malignancies, monoclonal gammopathy, autoimmune diseases, hepatitis C virus infection, diabetes mellitus, or chronic infections [2,3].

Clinically, FGN exhibits a broad spectrum of manifestations, ranging from asymptomatic proteinuria to nephrotic syndrome, nephritic syndrome, or even rapidly progressive glomerulonephritis (RPGN). The most common presentation is nephrotic syndrome, often accompanied by renal dysfunction and microscopic hematuria on urine sediment analysis. Notably, RPGN associated with FGN, although rare, represents a severe complication characterized by the rapid deterioration of renal function and an increased likelihood of requiring renal replacement therapy (RRT) [4].

The diagnosis of FGN relies on KB, integrating electron microscopy (EM) findings, traditionally the gold standard, with immunofluorescence (IF) and light microscopy (LM) data. LM reveals mesangial expansion and capillary wall thickening due to the accumulation of deposits, while the identification of the characteristic fibrillar nature of the deposits requires confirmation by EM. The histopathological presentation is variable, ranging from minimal alterations to mesangial expansion with a non-methenamine silver- and Congo red-negative material that typically extends peripherally, thickening the capillary walls and allowing differentiation from amyloidosis. The presence of crescents has been reported in 27% to 50% of cases [5], and approximately 4% may have a monoclonal origin.

IF typically demonstrates IgG deposition, predominantly of the IgG4 subclass, along with C3 deposits. A coarse and blurred staining pattern in the mesangium and capillary walls is characteristic; in some cases, deposits may be restricted to the capillary walls in a pseudolinear pattern, resembling that seen in anti-glomerular basement membrane (anti-GBM) antibody disease [6]. EM remains essential for confirming the fibrillary nature and size of the deposits, distinguishing FGN from other fibrillary diseases such as immunotactoid glomerulonephritis (ITGN), where fibrillary deposits exceed 30 nm in diameter.

More recently, the overexpression of DNA J homolog subfamily B member 9 (DNAJB9) has been identified in both idiopathic FGN and FGN associated with other conditions. More recently, the overexpression of DNA J homolog subfamily B member 9 (DNAJB9) has been identified in both idiopathic FGN and FGN associated with other conditions, including systemic lupus erythematosus (SLE), rheumatoid arthritis, hepatitis C virus infection, monoclonal gammopathy, diabetes mellitus, and chronic infections [7,8,9]. DNAJB9 positivity in fibrillary deposits, detectable via immunohistochemistry (IHC), has emerged as a valuable diagnostic tool. This marker can facilitate FGN diagnosis in the absence of EM or in cases where EM findings alone do not clearly differentiate amyloid, ITGN, or FGN based on fibril diameter or Congo red staining properties [7,8].

In patients with RPGN, rapid progression to acute kidney injury (AKI) may occur within weeks to months, underscoring the importance of early and accurate diagnosis. However, recognizing FGN remains challenging, as its clinical manifestations and laboratory findings can overlap with those of more common glomerulopathies, including IgA nephropathy (IgAN), membranoproliferative glomerulonephritis (MPGN), and even amyloidosis [8].

From a therapeutic perspective, there is no clear consensus on the optimal management of FGN. This is largely due to the scarcity of controlled clinical studies and the heterogeneous nature of the disease. Although various immunosuppressive strategies have been employed, their effectiveness remains variable and is often associated with significant adverse effects. The identification of specific biomarkers and the development of targeted therapies are priority areas for future research [10].

The prognosis of FGN varies depending on factors such as the severity of glomerular involvement, the presence of renal dysfunction at the time of diagnosis, and the response to treatment. In the absence of a standardized therapeutic approach, management is primarily supportive, focusing on symptom control and immunosuppressive therapies, including corticosteroids, cyclophosphamide, mycophenolate mofetil, and rituximab (RTX). However, the response to immunosuppressive therapy is often suboptimal, and a significant proportion of patients progress to end-stage renal disease (ESRD), ultimately requiring dialysis or kidney transplantation [8].

We present a case series of three patients with FGN, each exhibiting a distinct clinical course that underscores the heterogeneous nature of the disease. One patient developed RPGN secondary to FGN, a rare but clinically significant complication that highlights the potential aggressiveness of this condition. Below, we detail the clinical, histological, and therapeutic aspects of each case, emphasizing the diagnostic and treatment challenges associated with FGN.

## 2. Case Series

### 2.1. Case 1

A 39-year-old Caucasian woman with a history of well-controlled arterial hypertension (AHT) managed with an angiotensin II receptor blocker (ARB) and a thiazide diuretic. She had no family history of glomerulopathy and was referred from primary care to the nephrology outpatient clinic due to proteinuria, with a urinary protein excretion of 1.5 g/day. Serum creatinine (sCr) was 0.8 mg/dL. On physical examination, there was no evidence of hypertension or pretibial edema at the time of admission. Urinalysis revealed persistent microscopic hematuria (25–30 red blood cells per high-power field (RBC/HPF)). Titers of antinuclear antibodies (ANA), antineutrophil cytoplasmic antibodies (ANCAs), complement levels (C3 and C4), and immunoglobulins were within normal limits. Serological tests were negative for hepatitis B, hepatitis C, and human immunodeficiency virus (HIV). The remaining blood test results are summarized in Table 1.

To determine the etiology of renal involvement, a KB was performed. LM revealed 35 glomeruli, 8 of which were globally sclerosed. The remaining glomeruli exhibited capillary wall thickening and mesangial expansion, with three glomeruli showing segmental sclerotic lesions. The silver staining technique demonstrated a deflected pattern in both the basement membrane and the mesangium, with occasional areas of glomerular basement membrane (GBM) unfolding. Congo red and thioflavin stains were negative. Tubular atrophy and interstitial fibrosis were mild (<25%), and arteriolar hyalinosis was absent.

**Figure 1:**
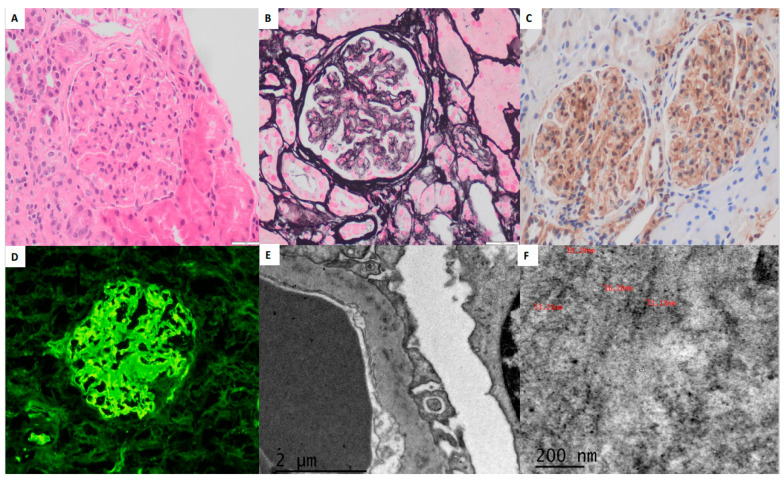
A glomerulus showing mild mesangial widening and thickening of the capillary walls (H&E, 40×) (**A**). Silver stain (40×) demonstrating non-argyrophilic deposits in the mesangial areas and capillary walls (**B**). DNAJB9 immunohistochemistry (20×) showing mesangial and capillary wall positivity (**C**). Immunofluorescence with IgG (40×) demonstrating intense deposits with a smudgy pattern (**D**). Electron microscopy (EM, 20,000×) revealing deposits within the glomerular basement membranes (**E**). At higher magnification, these deposits exhibit a fibrillary morphology with diameters ranging from 13 to 21 nm, consistent with FGN (**F**).

Direct immunofluorescence (DIF) performed on frozen tissue included 10 glomeruli, which showed smudgy mesangial and capillary wall deposits of IgG (+++/+++), C3 (++/+++), C4 (++/+++), C1q (++/+++), and both kappa and lambda light chains (+++/+++). IgA and IgM were negative. The glomeruli exhibited strong positivity for DNAJB9 immunostaining in the mesangial areas and capillary walls. EM revealed intramembranous and mesangial deposits composed of randomly arranged fibrils with a diameter of approximately 18–20 nm. Podocytes displayed extensive foot process effacement (Figure 1A–F). A diagnosis of FGN with a mesangial proliferative/sclerosing glomerulonephritis (MesGN) histologic pattern was established.

The patient underwent extensive malignancy screening, which was negative. Following a thorough discussion, one month after diagnosis, she was started on dapagliflozin 10 mg/day and rituximab (RTX) at a dose of 1 g intravenously (IV) twice, two weeks apart. After six months, proteinuria was significantly reduced to 150 mg/day, with microscopic hematuria decreasing to 5–10 RBC/HPF and stable sCr of 0.7 mg/dL, achieving complete remission (CR). It was decided to continue maintenance therapy with RTX at a dose of 1 g every six months for three years. No adverse effects related to RTX were observed. After three years, the patient remains in CR, with an sCr of 0.8 mg/dL, 5–10 RBC/HPF, and proteinuria of 250 mg/day.

### 2.2. Case 2

A 60-year-old Caucasian male with a history of well-controlled arterial hypertension (AHT), benign prostatic hypertrophy, and low-grade papillary urothelial carcinoma diagnosed four years prior. The carcinoma was treated with intravesical instillations of Bacillus Calmette-Guérin (BCG) with a favorable response and no evidence of tumor progression. He was referred to the nephrology department due to persistent proteinuria of 1.2 g/day despite maximized angiotensin II receptor blocker (ARB) therapy and microscopic hematuria (30–35 dysmorphic red blood cells per high-power field (RBC/HPF)).

Laboratory tests showed normal renal function (serum creatinine [sCr]: 0.7 mg/dL) and persistent hematuria on urinalysis. Serological testing was negative for hepatitis B, hepatitis C, and HIV. Autoimmune screening, including antinuclear antibodies (ANA), antineutrophil cytoplasmic antibodies (ANCA), cryoglobulins, complement levels (C3 and C4), and monoclonal protein testing, was also negative. The remaining blood test results are summarized in Table 1.

A KB specimen contained 20 glomeruli, 3 of which were globally sclerosed. Light microscopy (LM) revealed mesangial expansion along with increased rigidity and thickening of the glomerular basement membrane (GBM). Periodic acid–Schiff (PAS) staining was strongly positive in the mesangium and capillary walls, while silver staining demonstrated vacuolation and fraying of the capillary walls and mesangium. The interstitium showed minimal fibrosis and tubular atrophy, while the arteries exhibited mild arteriosclerosis, and the arterioles displayed mild hyalinization. Congo red staining was negative.

**Figure 2:**
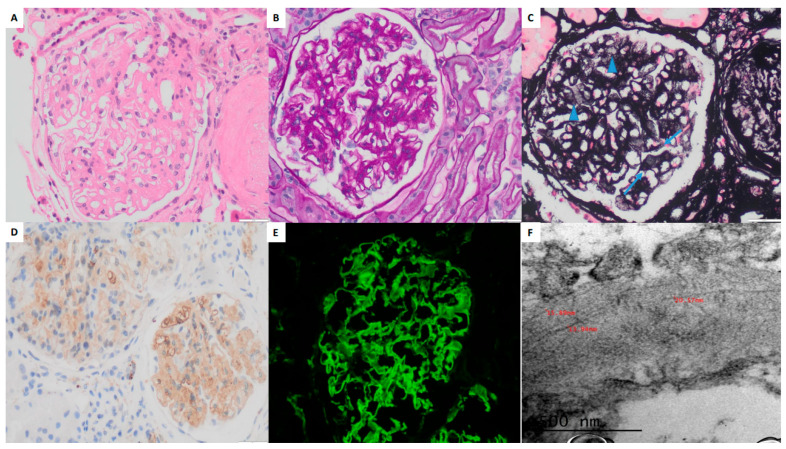
Light microscopy findings showing mesangial expansion and thickening of the capillary walls (H&E, 40×) (**A**). PAS stain (40×) demonstrating mesangial widening (**B**). Silver stain (40×) revealing expansion of the mesangial regions by non-argyrophilic material (arrowheads) and small holes in the basement membranes (arrows) (**C**). DNAJB9 immunohistochemistry (20×) demonstrating global and diffuse staining in the mesangium and capillary walls (**D**). Immunofluorescence with IgG (40×) revealing deposits in a mesangial and capillary wall pattern (**E**). Electron microscopy (EM, 50,000×) showing fibrillary deposits in the basement membrane, with diameters ranging from 13 to 20 nm (**F**).

Direct immunofluorescence (DIF) performed on frozen tissue revealed a gross pseudolinear staining pattern for IgG (+++/+++), IgM (++/+++), kappa and lambda light chains with equal intensity and distribution (+/+++), C3 (+++/+++), C4 (++/+++), and C1q (+/++/+++) in both the capillary walls and mesangium. Immunohistochemistry (IHC) demonstrated intense DNAJB9 positivity in the mesangial and capillary loops. Electron microscopy (EM) identified non-branching, randomly arranged fibrillar deposits measuring approximately 15–20 nm in diameter within the mesangium and GBM. Podocytes exhibited extensive foot process effacement (Figure 2A–F). A diagnosis of FGN with a mesangial proliferative/sclerosing glomerulonephritis (MesGN) histologic pattern was established.

The patient was started on losartan potassium 100 mg/day, dapagliflozin 10 mg/day, and protein and sodium restriction. Ten days after diagnosis, he was initiated on rituximab (RTX) at a dose of 375 mg/m^2^ IV weekly for four weeks. After six months, proteinuria decreased slightly to 920 mg/day while renal function remained stable (sCr: 0.8 mg/dL). Antiproteinuric therapy was intensified with the addition of spironolactone (50 mg/day), and RTX maintenance therapy was continued at 1 g every six months. Two years after his kidney biopsy, the patient maintains stable renal function with a urine protein excretion of 840 mg/day and Cr 1.1 mg/dL.

### 2.3. Case 3

**Figure 3:**
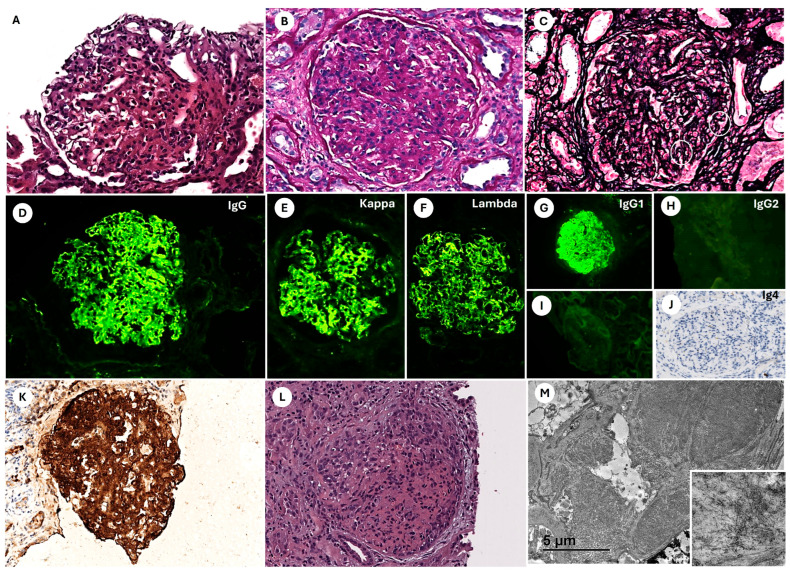
First biopsy (**A**–**K**) demonstrating mesangial expansion and proliferation (**A**) with PAS-positive deposits (**B**) and double contours in capillary loops ((**C**), white circles), consistent with a membranoproliferative glomerulonephritis pattern. Direct immunofluorescence showing positivity for IgG (**D**) with coexpression of kappa (**E**) and lambda (**F**) light chains, along with exclusive positivity for IgG1 (**G**–**J**). Immunohistochemistry revealing intense and diffuse DNAJB9 positivity (**K**). Second biopsy showing mesangial proliferation and expansion associated with focal crescent formation (**L**). Transmission electron microscopy (**M**) displaying massive electron-dense mesangial and subendothelial fibrillar deposits, randomly arranged (lower right square). Stains and magnifications: H&E (**A**,**L**), PAS (**B**), methenamine silver (**C**), direct immunofluorescence for IgG (**D**), kappa (**E**), lambda (**F**), IgG1 (**G**), IgG2 (**H**), IgG3 (**I**), immunoperoxidase for IgG4 (**J**), and DNAJB9 (**K**) at 20× magnification. Transmission electron microscopy at 500× (**M**) and 2500× (lower square).

A 71-year-old male with a history of chronic kidney disease (CKD) stage IIIa/A3 (sCr: 1.35 mg/dL, CKD Epidemiology Collaboration (CKD-EPI)-2021: 52 mL/min/1.73 m^2^, proteinuria: 859 mg/day, without microhematuria), most likely secondary to diabetic kidney disease (DKD), arterial hypertension (AHT), and type 2 diabetes mellitus (T2DM). His medication included amlodipine, olmesartan, hydrochlorothiazide, empagliflozin, metformin, and pantoprazole. Four months later, during a follow-up nephrology consultation, worsening renal function was observed, with an increase in sCr to 2.6 mg/dL, proteinuria rising to 3.2 g/day, and newly detected hematuria (35 RBC/HPF) on urinalysis. Given the clinical course, which was not compatible with the expected progression of DKD, further investigations were conducted to identify other potential causes of kidney deterioration. Serological tests were negative for hepatitis B, hepatitis C, and HIV. Additionally, ANA and ANCA were negative, and complement levels (C3 and C4) were within normal limits. Since no clear etiology for the renal dysfunction was identified, a kidney biopsy was performed. LM revealed 23 glomeruli, 6 of which were globally sclerosed. The viable glomeruli exhibited global and diffuse hypertrophy with marked mesangial expansion due to an increase in matrix and mesangial cellularity. PAS staining was positive, while methenamine silver staining was negative. The capillary loops were diffusely thickened, displaying PAS-positive and silver-negative deposits, frequently forming double contour images. Capillary lumina were occupied by endocapillary hypercellularity. Congo red staining showed red (congophilic) positivity at the glomerular level, but birefringence under polarized light was negative. DIF performed on frozen tissue revealed IgG (++), C3 (+++), C1q (++), and kappa (+++) and lambda (+++) light chains in a thick granular, homogeneous staining pattern within the capillary loops and mesangium. IHC demonstrated strong C4d and DNAJB9 positivity in the mesangial regions and capillary loops. EM showed randomly arranged, non-branching fibrillar deposits with diameters of approximately 13–20 nm within the mesangium and basement membranes (Figure 3A–K). Findings were consistent with fibrillary glomerulonephritis (FGN) with a membranoproliferative glomerulonephritis (MPGN) pattern. Given the patient’s age at diagnosis, an extensive malignancy workup was performed, including thoracoabdominal–pelvic computed tomography with contrast, as well as upper and lower endoscopy, all of which were negative. The patient was followed as an outpatient; however, one month after the biopsy, renal function deteriorated further, with sCr increasing to 7.39 mg/dL. He developed 2+/4+ lower limb edema and nephrotic syndrome, with proteinuria rising to 6.4 g/day and serum albumin decreasing to 2.4 g/dL. Given the rapid deterioration, the patient was hospitalized for a second kidney biopsy. Serological, autoimmune, and cryoglobulin studies were repeated, and all remained negative. Complement levels were normal. Serum immunofixation did not detect monoclonal immunoglobulin. A renal ultrasound revealed increased echogenicity in both kidneys. A second KB was performed. LM showed 43 glomeruli, of which 40% exhibited extracapillary proliferation, all in the cellular stage, with crescent formation. DIF revealed IgG (+++), C3 (++), C1q (+), and C4 (+) with a granular mesangial and capillary wall staining pattern. No restriction for light chains was observed (kappa +++/lambda +++). EM confirmed the persistence of fibrillar deposits characteristic of FGN (Figure 3L–M). The findings were consistent with FGN associated with focal extracapillary proliferation (crescentic GN). The patient was initiated on hemodialysis for uremia management. Given the biopsy findings of crescentic GN associated with FGN, he started on intravenous (IV) pulse corticosteroid therapy (methylprednisolone 250 mg/day for 3 days), followed by daily oral prednisone (1 mg/kg) with a tapering regimen over six months. In addition, the patient was treated with cyclophosphamide. After six months, the patient remained dialysis-dependent despite completing six cycles of cyclophosphamide in combination with corticosteroid therapy. Subsequently, he started on rituximab (RTX) at a dose of 1 g biannually. However, one year after the initial KB, due to the absence of renal function recovery, it was decided to discontinue immunosuppressive therapy (RTX).

## 3. Discussion

We present a series of three cases of FGN, in which we review the clinical, histopathological, and therapeutic data, as well as the evolution of each case. FGN is an immune complex-mediated disease that is idiopathic in most cases and is a rare entity but when diagnosed quickly. Diagnosis primarily relies on histopathological findings, with the identification of non-amyloid fibrils that are randomly arranged, unbranched, and approximately 20 nm in diameter on EM, serving as its hallmark [8]. It is a rare condition, accounting for less than 1% of kidney biopsies, yet it has a significant clinical impact due to its high progression rate to end-stage renal disease (ESRD) [8].

FGN was first described by Rosenmann and Eliakim, who reported a case of nephrotic syndrome associated with the deposition of amyloid-like material [11]. In 1987, Alpers et al. [12] reported eight cases of patients with renal dysfunction, most of whom presented with nephrotic-range proteinuria. They coined the term “fibrillary nephritis” and characterized the fibrils as unbranched, randomly arranged structures in the mesangium and capillary walls, measuring approximately 20 nm in diameter.

The precise pathophysiology of FGN remains uncertain. However, there is evidence suggesting a genetic predisposition to the disease [13]. FGN is an immune complex glomerulonephritis (GN), where immune complexes polymerize into fibrils due to their relative homogeneity, possibly related to the frequent IgG4 subclass restriction observed in FGN. In 2018, a better understanding of FGN was achieved when DNAJB9 was identified as being overexpressed in FGN glomeruli. DNAJB9 functions as a cochaperone to binding immunoglobulin protein, assisting in protein folding and the degradation of misfolded proteins, a process termed the “unfolded protein response.” DNAJB9 was shown to colocalize with IgG and components of the classical complement pathway in glomeruli, supporting the theory that it may act as an autoantigen in FGN, serving as a precursor that traps immunoglobulins and triggers an autoimmune response. An alternative theory suggests that DNAJB9 is not an autoantigen but rather a binding protein for misfolded IgG molecules, leading to their aggregation and deposition in the kidney as non-amyloid fibrils. Neither of these two theories has been confirmed to date [13]. It is likely that the production of DNAJB9 occurs outside the kidney, as the disease tends to recur in allografts and evidence of fibril deposition has been found in extrarenal organs; however, the exact site of production remains unknown [13]. While DNAJB9 staining assists diagnostic accuracy in FGN, staining levels do not so far correlate with clinical responses to rituximab or renal outcomes, suggesting the need for additional studies or that DNAJB9 use could be primarily as a diagnostic marker, at least until further validation as a prognostic or therapeutic indicator is accomplished.

In our series, all cases were presented with microscopic hematuria. Schober et al. [14] reported a series of 42 patients with FGN, of whom 95% had hematuria. Additionally, the three patients of our series had proteinuria, with one meeting the criteria for nephrotic syndrome. Rosenstock et al. [2] reported a series of 61 patients with FGN, all of whom presented with proteinuria, and 52% had nephrotic syndrome at the time of diagnosis. In our series, only one of the three patients had renal dysfunction, which differs from the findings in the literature. Andeen et al. [3] reported a series of 66 patients with FGN, in which over 80% had renal dysfunction at presentation. All three patients in our series had a history of hypertension, consistent with findings by Gambella et al. [7], who reported a cohort of 77 patients with FGN, in which hypertension was the most common clinical condition at diagnosis (34 cases).

Patient 1 of our series did not have any underlying diagnosis likely to be pathogenetically associated with the development of FGN; however, patient 2 and patient 3 had medical histories that could potentially be related to FGN. Patient 2 had a history of urothelial carcinoma. Malignant neoplasms, more frequently solid tumors than hematologic malignancies, have been reported in 4% to 23% of patients with FGN, with a wide variety of cancer types observed and no clear predilection for any specific type. The diagnosis of cancer may precede or follow the diagnosis of FGN [5]. There is ongoing debate as to whether FGN represents a true paraneoplastic phenomenon or whether the increased prevalence of cancer is solely attributable to the advanced median age of FGN patients.

Patient 3 was diagnosed with T2DM, which is reported in 5.6% to 28% of patients with FGN. A possible association between T2DM and FGN has been proposed, based on the concept that accelerated protein glycosylation in diabetics and the accumulation of advanced glycation end products could cross-link with other structural and circulating proteins, thereby predisposing individuals to the development of FGN; however, this hypothesis has not yet been confirmed [15].

The case of patient 3 underscores the importance of early diagnosis and aggressive treatment in rapidly progressive forms. The literature indicates that patients with RPGN have poorer outcomes due to the presence of crescents and fibrinoid necrosis in over 50% of glomeruli, as observed in this case [16]. Studies by Hogan et al. emphasize that a combination of cyclophosphamide and corticosteroids remains the cornerstone of treatment in such severe cases, although the risk of progression to ESRD remains high [17].

In contrast, patients 1 and 2 had more favorable outcomes. Both responded positively to rituximab treatment, consistent with findings by Attieh et al. [8], who reported that patients with preserved renal function and a lower fibrillar burden achieved better clinical outcomes. This highlights the importance of considering rituximab as a therapeutic option in selected patients despite the lack of standardized guidelines [18]. However, studies by Hogan et al. and Andeen et al. reported that up to 50% of patients with FGN progress to ESRD within an average of 2–4 years. While kidney transplantation is a viable option, graft recurrence poses a significant challenge, emphasizing the need for novel therapeutic strategies [17,18].

FGN can present with various histopathological patterns on light microscopy (LM), ranging from mesangial expansion without hypercellularity (MesGN) to a membranoproliferative glomerulonephritis (MPGN) pattern. Congo red staining is typically negative [19]. In our three cases, electron microscopy (EM) revealed mesangial expansion and capillary wall thickening. Patient 3 exhibited an MPGN pattern of glomerular injury, eventually progressing to crescentic glomerulonephritis (GN). Additionally, this patient demonstrated Congo red positivity, a finding observed in approximately 5% of FGN cases. In general, the basis for congophilia in congophilic FGN remains unknown. However, considering the resemblance of the fibrillary quaternary structure between FGN and amyloid deposits observed by transmission EM, it is conceivable that FGN and amyloid fibrils may share certain similarities in their secondary structure [20]. Alexander et al. described a case series of 18 patients with Congo red-positive FGN, reporting that these patients frequently had underlying conditions, particularly monoclonal gammopathy and hepatitis C virus (HCV) infection [21]. In patient 3, the presence of congophilia prompted an extensive search for an underlying explanation, which remained inconclusive despite thorough investigation ruling out the following: (1) monoclonal gammopathy, (2) infection, and (3) malignancy. Thus, in our patient 3, the coexistence of congophilia and the clinical course of RPGN appears to be casual rather than causal.

The most frequent IF findings in FGN patients are capillary wall and mesangial staining for polyclonal IgG and C3, with or without C1q. Most cases exhibit IgG4 dominance or IgG4 and IgG1 codominance within the IgG subclass [22]. In our series, all three cases were positive for IgG, C3, C4, C1q, kappa, and lambda by IF. Nasr et al. reported a series of 66 FGN patients, in which 100%, 92%, 60%, 85%, and 90% of cases were positive for IgG, C3, C1q, kappa, and lambda, respectively [5]. Gambella et al., in their series of 77 patients, had appropriate material for expression evaluation in 67 cases for IgG, 66 for C3, and 55 for light and heavy chains [7]. Andeen et al. reported that only 6% of cases exhibited monoclonal staining (one light chain positive [κ or λ] with absent staining for the other) on IF [13].

In our series, immunohistochemistry (IHC) demonstrated strong DNAJB9 positivity in all three cases, localized to the mesangium and capillary loops. DNAJB9-IHC staining has a reported sensitivity of 98% and specificity of 99% as a histological marker for FGN. DNAJB9 biomarker identification represents a major advancement in diagnosing FGN, revolutionizing its differentiation from other fibrillary glomerulopathies. DNAJB9 exhibits near-perfect sensitivity and specificity, facilitating early and accurate diagnosis. Furthermore, its potential role in the disease’s pathogenesis may provide opportunities for targeted therapies in the future [19]. This staining is useful in distinguishing Congo red-positive FGN from amyloidosis and differentiating FGN from other conditions such as diabetic fibrillosis and collagen-fibrin glomerulopathy [23]. In Gambella et al.’s series, DNAJB9 was detected in 73 of the 74 available cases of FGN, with predominantly homogeneous and strong positivity in 68 cases, while 5 cases exhibited moderate and scattered expression in mesangial areas and occasionally in the glomerular basement membrane (GBM) [7].

EM findings in FGN typically include randomly oriented, straight, non-branching fibrils measuring approximately 20 nm in diameter (range: 12–24 nm), primarily deposited within the mesangium and occasionally extending along the GBM. Podocyte foot process effacement is frequently observed in regions with fibril accumulation [24]. In our series, all three cases demonstrated non-branching fibrillar deposits, randomly arranged and measuring up to approximately 20 nm in diameter, within the mesangium and basement membranes. Additionally, extensive podocyte foot process effacement was observed. Javaugue et al. reported a series of 27 FGN patients, where EM revealed extracellular fibrils predominantly within the mesangium and subepithelial regions, with occasional extension into the dense lamina of the GBM. The mean fibril diameter was 15.4 nm (range: 7–26 nm) [25]. Similarly, Alexander et al. described 18 FGN cases in which all patients exhibited glomerular deposits of non-branching, randomly oriented fibrils affecting the mesangium (100% of cases) and GBM (17 cases). The fibril size was measured in 15 cases, with a mean diameter of 14 nm (range: 11–18 nm) [21].

FGN and immunotactoid glomerulonephritis (ITGN) share clinical and pathological features but are distinct entities. FGN is characterized by fibrillar deposits measuring 12–24 nm in diameter, whereas ITGN deposits are microtubular, larger (15–50 nm), and exhibit a different structural organization [23]. Clinically, ITGN is more strongly associated with B-cell dyscrasias such as multiple myeloma or chronic lymphocytic leukemia, whereas FGN is predominantly idiopathic [19,23]. The differential diagnosis is further aided by DNAJB9 staining, which is positive in FGN but negative in ITGN, enabling precise differentiation even in the absence of electron microscopy [23]. These differences highlight the importance of integrating advanced diagnostic tools into the clinical management of these conditions.

Regarding treatment, there are no prospective randomized controlled trials for FGN, and currently, no standardized therapeutic guidelines exist. Despite the lack of conclusive evidence supporting the benefit of immunosuppression in slowing renal progression, its use remains common. Various therapeutic strategies have been evaluated, ranging from corticosteroid monotherapy to combinations with cyclophosphamide, mycophenolate mofetil, rituximab (RTX), or calcineurin inhibitors (CNIs), with heterogeneous results. Few studies have described a beneficial impact on the disease course with steroids alone; as described by Dickenmann et al., three patients treated with prednisone showed an impressive reduction in proteinuria, which completely remitted in two cases, and no signs of declining renal function during the observation period [26]. High-dose steroids and cyclophosphamide have shown relative success in some isolated case reports, mainly in cases of FGN with MPGN pattern, especially if there are crescents, as reported by Mahajan et al. [27] and Blume et al. [28], who described the cases of a patient with FGN MPGN and another with necrotizing FGN with good therapeutic response, respectively

Given that glomerular deposits in FGN consist of polyclonal IgG, some authors have proposed that FGN represents an autoimmune disease that may respond to RTX, a monoclonal anti-CD20 antibody [29]. In a study by Hogan et al. involving 12 FGN patients with a median baseline creatinine of 2.1 mg/dL and proteinuria of 4497 mg/day, RTX treatment was associated with no disease progression in 33% of patients after 38 months of follow-up [17]. Similarly, Javaugue et al. reported long-term follow-up of 27 FGN patients, of whom 7 received RTX as part of their treatment regimen. One patient achieved complete renal remission, four achieved partial remission, and two had no response to RTX therapy. The median estimated glomerular filtration rate (eGFR) before treatment among the five responders was 77 mL/min/1.73 m^2^ (range: 35–96 mL/min/1.73 m^2^) [25]. On the other hand, the absence of predictive biomarkers to identify patients who may benefit from immunosuppression further complicates treatment decisions, leaving the therapeutic approach dependent on clinical severity and individual histologic features. In our series, all three patients received renin–angiotensin–aldosterone system (RAAS) blockade and sodium–glucose cotransporter-2 (SGLT2) inhibitors. Patients 1 and 2 were treated with rituximab (RTX) shortly after diagnosis and continued therapy according to the prescribed regimen for three years, maintaining adequate renal function and proteinuria control. These positive outcomes may be related to their preserved renal function and the short time interval between diagnosis and the initiation of RTX. This aligns with findings from Hogan et al., who reported that patients who did not progress to ESRD had lower serum creatinine levels, higher eGFR, and a shorter median duration from diagnosis to treatment compared to those who progressed [17].

Conversely, patient 3, who presented with an MPGN-pattern FGN and did not receive RTX early, subsequently developed RPGN and remains dialysis-dependent despite treatment with cyclophosphamide, corticosteroids, and later RTX. While RTX has emerged as a promising therapy, its efficacy in patients with advanced renal dysfunction remains questionable. This supports Hogan’s hypothesis that RTX may only prevent or delay disease progression in patients with preserved renal function and may not be effective beyond a “point of no return” in renal dysfunction [17]. Furthermore, the MPGN pattern itself represents an adverse prognostic factor, as it is associated with higher serum creatinine levels, greater proteinuria, and a shorter median time to ESRD, along with sclerosing histological patterns [30]. Recently, Erikson et al. [30] conducted a prospective pilot clinical trial in which 11 patients with idiopathic FGN received two cycles of RTX (1 g each) administered two weeks apart initially and then repeated at six months. The primary outcome was defined as preservation of renal function at 12 months, indicated by stable or increased creatinine clearance (CrCl). The secondary outcome included achieving complete remission (CR), defined as proteinuria <300 mg/24 h, or partial remission (PR), defined as proteinuria <3 g/24 h with at least a 50% reduction in proteinuria. Serum DNAJB9 levels were also measured at baseline and at 6 and 12 months. CrCl remained stable during follow-up, from 47.7 mL/min/1.73 m^2^ at baseline to 43.7 mL/min/1.73 m^2^ at 12 months (P = 0.15). Proteinuria decreased from 4.43 g/24 h (range: 1.6–5.53 g/24 h) at baseline to 1.9 g/24 h (range: 0.46–5.26 g/24 h) at 12 months, but the reduction did not reach statistical significance (P = 0.06). None of the patients achieved CR, while 3 of 11 achieved PR. Additionally, no significant changes in DNAJB9 levels were observed following RTX treatment. The study concluded that treatment with two cycles of RTX over six months was associated with stabilization of renal function but did not significantly improve renal function or proteinuria.

Currently, two clinical trials are underway to evaluate the efficacy of anti-CD20 therapy in FGN. The first study (NTC06680349), an Italian trial, is assessing the clinical and histological effects of two RTX-based regimens in DNAJB9-positive FGN. It compares RTX monotherapy (375 mg/m^2^ every four weeks) with intensive B-cell depletion therapy (IBCDT), which includes RTX (375 mg/m^2^ every four weeks followed by two additional doses at one and two months), cyclophosphamide (two 10 mg/kg pulses, adjusted for renal function, on days 4 and 17), and methylprednisolone (three bolus doses of 15 mg/kg) followed by oral prednisone (initial dose of 50 mg, gradually tapered over four months). The primary outcome will be the mean renal response at three and six months, defined as CR (proteinuria reduction to <0.5 g/day with stable renal function [<20% increase in serum creatinine]) or PR (≥50% reduction in proteinuria with stable renal function). Recruitment was completed in November 2024, with results pending. The second study, currently in the recruitment phase, aims to determine the efficacy and safety of obinutuzumab in the treatment of FGN. It is a single-center, phase 2, open-label trial evaluating obinutuzumab (NTC06295770). Patients with biopsy-proven FGN, proteinuria >1 g/24 h, and eGFR ≥20 mL/min/1.73 m^2^ will receive obinutuzumab at a dose of 1 g IV on day 1 and 1 g IV on day 15, followed by a second identical cycle at month 6. The primary outcome will be changes in proteinuria from baseline to 12 months post-treatment. Secondary outcomes include proteinuria reduction at six months, CR rate (proteinuria <0.5 g/24 h with no more than a 20% decrease in eGFR), PR rate (≥50% proteinuria reduction with proteinuria <3.5 g/24 h and no more than a 20% decrease in eGFR), improvement in serum albumin levels, stabilization of renal function (defined as no more than a 20% decrease in eGFR at six and 12 months), and the incidence of serious adverse events (SAEs), including severe infections such as urinary tract infections (UTIs), pyelonephritis, pneumonia, or other systemic infections requiring hospitalization.

The prognosis of FGN remains poor, with up to 50% of patients progressing to ESRD within two years of diagnosis [31]. In cases where renal replacement therapy (RRT) becomes necessary, kidney transplantation may be a viable option; however, the risk of recurrence remains a significant concern [32]. Although the pathophysiology of FGN is not entirely understood, a link between DNAJB9 and fibril formation was suggested [33]. The development of targeted therapies aimed at inhibiting DNAJB9 formation or binding, tested in prospective controlled trials, could potentially alter the disease course.

## 4. Conclusions

In conclusion, this case series underscores the need for personalized therapeutic approaches and early diagnosis to improve the prognosis of patients with FGN. Recent advances, particularly the identification of the DNAJB9 biomarker, have significantly enhanced diagnostic accuracy and may pave the way for targeted therapies in the future. A deeper understanding of the pathophysiological mechanisms, prognostic factors, and therapeutic options for FGN could have a substantial impact on patient outcomes.

## Figures and Tables

**Table 1:** Baseline laboratory characteristics (at diagnosis) of patients 1 and 2 with fibrillary GN and of patient 3 at the time of the second renal biopsy.

	Case 1	Case 2	Case 3	Normal Values—Unit
WBC	9.43	8.36	10.35	4–10 × 10^3^/µL
Hb	13.6	16.2	9.8	12–16 g/dL
Platelet count	285	209	268	150–450 × 10^3^/µL
Reticulocyte count	2.7	2	3	2–4%
LDH	385	287	304	120–246 U/L
Coombs test	Negative	Negative	Negative	NA
Total bilirubin	0.3	0.8	0.28	0.2–1.3 mg/dL
Indirect bilirubin	0.15	0.3	0.10	0–1.1 mg/dL
Total protein	6.1	5.9	4.9	6.4–8.7 g/dL
Serum albumin	3.34	3.2	2.4	3–5.5 g/dL
GOT	15	38	20	5–32 IU/L
GPT	9	25	10	5–33 IU/L
Serum urea	25	29	225	17–60 mg/dL
Serum creatinine	0.8	0.71	7.39	0.7–1.2 mg/dL
Serum sodium	141	140	127	135–145 mmol/L
Serum potassium	4.5	4.1	4.7	3.5–5.5 mmol/L
Serum clorum	107	106	105	95–110 mmol/L
CRP	0.06	0.15	3.48	0.1–0.5 mg/dL
HbsAg	Negative	Negative	Negative	NA
HCV-Ab	Negative	Negative	Negative	NA
HIV	Negative	Negative	Negative	NA
Autoantibodies CFH	Negative	Negative	Negative	<18 AU/L
C3 nephritic factor	Negative	Negative	Negative	Ratio > 1.022
C3	90	98	87	90–180 mg/dL
C4	24.3	26	23.8	10–40 mg/dL
RF	Negative	Negative	Negative	<15 IU/ml
ANA, anti-ds-DNA, ANCA, and cryoglobulin	Negative	Negative	Negative	NA
Anti-GBM	Negative	Negative	Negative	<1 AI
Anti-PLA2R Ab (ELISA)	Negative	Negative	Negative	NA
IgG	1500	780	822	800–1600 mg/dL
IgA	100	345	226	70–400 mg/dL
IgM	135	114	43.8	90–180 mg/dL
β2-microglobulin	0.20	0.19	1.64	0–20 mg/dL
24 h urine total protein excretion	1.5	1.2	8.4	<0.15 g/24 h
Urine red blood cells	25–30	30–35	50–100	RBC/HPF
SPEP/SIFE	Polyclonal	Polyclonal	Polyclonal	NA
UPEPE/UIFE	Negative	Negative	Negative	NA
FLC κ	9	13.20	61.6	4.90–13.70 mg/L
FLC λ	10	18.20	47	7.60–19.50 mg/L
FLC κ/λ	0.9	0.73	1.31	0.27–1.67

NA: not applicable, WBC: white blood cells, Hb: hemoglobin, LDH: lactate dehydrogenase, GOT: glutamate oxaloacetate transaminase, GPT: glutamate pyruvate transaminase, CRP: C-reactive protein, HBsAg: hepatitis B surface antigen, HCV-Ab: hepatitis C virus antibody; HIV: human immunodeficiency virus; CFH: complement factor H; C3: complement 3; C4: complement 4, RF: rheumatoid factor, ANA: antinuclear antibody, Antids-DNA: anti-double-stranded DNA antibody, ANCA: anti-neutrophil cytoplasmic autoantibody, Anti-PLA2R Ab (ELISA): Anti-Phospholipase A2 Receptor Antibody (Enzyme-Linked ImmunoSorbent Assay), Ig: immunoglobulin, SPEP: serum protein electrophoresis, SIFE: serum immunofixation electrophoresis, UPEP/UIFE: urine protein electrophoresis/urine immunofixation electrophoresis, FLC: free light chain, κ: kappa, λ: lambda, RBC/HPF: red blood cell/high-power field.

## Data Availability

No new data were created or analyzed in this study. The data used to support the findings of this study are available from the corresponding author on request (contact J.C.D.L.F., jflomer@mde.es).

## References

[1] Nakhoul G.N., Simon J.F. (2016). Fibrillary glomerulonephritis associated with limited scleroderma: A case report. Clin. Nephrol..

[2] Rosenstock J.L., Markowitz G.S., Valeri A.M., Sacchi G., Appel G.B., D’Agati V.D. (2003). Fibrillary and immunotactoid glomerulonephritis: Distinct entities with different clinical and pathologic features. Kidney Int..

[3] Andeen N.K., Troxell M.L., Riazy M., Avasare R.S., Lapasia J., Jefferson J.A., Akilesh S., Najafian B., Nicosia R.F., Alpers C.E. (2019). Fibrillary Glomerulonephritis: Clinicopathologic Features and Atypical Cases from a Multi-Institutional Cohort. Clin. J. Am. Soc. Nephrol..

[4] Mallett A., Tang W., Hart G., McDonald S.P., Hawley C.M., Badve S.V., Boudville N., Brown F.G., Campbell S.B., Clayton P.A. (2015). End-Stage Kidney Disease Due to Fibrillary Glomerulonephritis and Immunotactoid Glomerulopathy—Outcomes in 66 Consecutive ANZDATA Registry Cases. Am. J. Nephrol..

[5] Nasr S.H., Valeri A.M., Fervenza F.C. (2011). Fibrillary glomerulonephritis: A report of 66 cases from a single institution. Clin. J. Am. Soc. Nephrol..

[6] Jennette J.C., Olson J.L., Silva F.G., D’Agati V.D. (2014). Heptinstall Pathology of the Kidney.

[7] Gambella A., Pitino C., Barreca A., Nocifora A., Giarin M.M., Bertero L., Biancone L., Roccatello D., Papotti M., Cassoni P. (2022). DNAJB9 is a Reliable Immunohistochemical Marker of Fibrillary Glomerulonephritis: Evaluation of Diagnostic Efficacy in a Large Series of Kidney Biopsies. Biomedicines.

[8] Attieh R.M., Yang Y., Rosenstock J.L. (2024). Updates on the Diagnosis and Management of Fibrillary Glomerulonephritis. Adv. Kidney Dis. Health.

[9] Klomjit N., Alexander M.P., Zand L. (2020). Fibrillary Glomerulonephritis and Dnaj Homolog Subfamily B Member 9 (DNAJB9). Kidney360.

[10] Lusco M.A., Fogo A.B., Najafian B., Alpers C.E. (2015). AJKD Atlas of Renal Pathology: Fibrillary Glomerulonephritis. Am. J. Kidney Dis..

[11] Rosenmann E., Eliakim M. (1977). Nephrotic syndrome associated with amyloid-like glomerular deposits. Nephron.

[12] Alpers C.E., Rennke H.G., Hopper J., Biava C.G. (1987). Fibrillary glomerulonephritis: An entity with unusual immunofluorescence features. Kidney Int..

[13] Andeen N.K., Yang H.Y., Dai D.F., MacCoss M.J., Smith K.D. (2018). DnaJ homolog subfamily B member 9 is a putative autoantigen in fibrillary GN. J. Am. Soc. Nephrol..

[14] Schober F.P., Jobson M.A., Poulton C.J., Singh H.K., Nickeleit V., Falk R.J., Jennette J.C., Nachman P.H., Iii W.F.P. (2017). Clinical Features and Outcomes of a Racially Diverse Population with Fibrillary Glomerulonephritis. Am. J. Nephrol..

[15] González-Cabrera F., Henríquez-Palop F., Ramírez-Puga A., Santana-Estupiñán R., Plaza-Toledano C., Antón-Pérez G., Marrero-Robayna S., Ramírez-Medina D., Gallego-Samper R., Vega-Díaz N. (2013). The occurrence or fibrillary glomerulonephritis in patients with diabetes mellitus may not be coincidental: A report of four cases. Case Rep. Med..

[16] Raikar M., Shafiq A. (2022). Fibrillary Glomerulonephritis: A Great Mimicker of Rapidly Progressive Glomerulonephritis. Cureus.

[17] Hogan J., Restivo M., Canetta P.A., Herlitz L.C., Radhakrishnan J., Appel G.B., Bomback A.S. (2014). Rituximab treatment for fibrillary glomerulonephritis. Nephrol. Dial. Transplant..

[18] Andeen N.K., Kung V.L., Robertson J., Gurley S.B., Avasare R.S., Sitaraman S. (2022). Fibrillary glomerulonephritis, DNAJB9, and the unfolded protein response. Glomerular Dis..

[19] Herrera G.A., Turbat-Herrera E.A. (2010). Renal diseases with organized deposits: An algorithmic approach to classification and clinicopathologic diagnosis. Arch. Pathol. Lab. Med..

[20] Puchtler H., Sweat F., Levine M. (1962). On the binding of Congo red by amyloid. J. Histochem. Cytochem..

[21] Alexander M.P., Dasari S., Vrana J.A., Riopel J., Valeri A.M., Markowitz G.S., Hever A., Bijol V., Larsen C.P., Cornell L.D. (2018). Congophilic fibrillary glomerulonephritis: A case series. Am. J. Kidney Dis..

[22] Sethi S., Adeyi O.A., Rennke H.G. (2001). A case of fibrillary glomerulonephritis with linear immunoglobulin G staining of the glomerular capillary walls. Arch. Pathol. Lab. Med..

[23] Nasr S.H., Sirac C., Bridoux F., Javaugue V., Bender S., Rinsant A., Kaaki S., Pinault E., Dasari S., Alexander M.P. (2019). Heavy Chain Fibrillary Glomerulonephritis: A Case Report. Am. J. Kidney Dis..

[24] Cantillo J.J., López R.D.P., Andrade R.E. (2009). Enfermedad de depósito glomerular: A propósito de un caso de glomerulonefritis fibrilar. Biomedica.

[25] Javaugue V., Karras A., Glowacki F., McGregor B., Lacombe C., Goujon J.-M., Ragot S., Aucouturier P., Touchard G., Bridoux F. (2013). Long-term kidney disease outcomes in fibrillary glomerulonephritis: A case series of 27 patients. Am. J. Kidney Dis..

[26] Dickenmann M., Schaub S., Nickeleit V., Mihatsch M., Steiger J., Brunner F. (2002). Fi-brillary glomerulonephritis: Early diagnosis associated with steroid responsiveness. Am. J. Kidney Dis..

[27] Mahajan S., Kalra V., Dinda A.K., Tiwari S.C., Agarwal S.K., Bhowmik D., Dash S.C. (2005). Fibrillary glomerulonephritis presenting as rapidly progressive renal failure in a young female: A case report. Int. Urol. Nephrol..

[28] Blume C., Ivens K., May P., Helmchen U., Jehle P.M., Riedel M., Keller F., Gra-bensee B. (2002). Fibrillary glomerulonephritis associated with crescents as a therapeutic challenge. Am. J. Kidney Dis..

[29] Bridoux F., Hugue V., Coldefy O., Goujon J.-M., Bauwens M., Sechet A., Preud’Homme J.-L., Touchard G. (2002). Fibrillary glomerulonephritis and immunotactoid (microtubular) glomerulopathy are associated with distinct immunologic features. Kidney Int..

[30] Erickson S.B., Zand L., Nasr S.H., Alexander M.P., Leung N., Drosou M.E., Fervenza F.C. (2021). Treatment of fibrillary glomerulonephritis with rituximab: A 12-month pilot study. Nephrol. Dial. Transplant..

[31] Motwani S.S., Herlitz L., Monga D., Jhaveri K.D., Lam A.Q., American Society of Nephrology Onco-Nephrology Forum (2016). Paraprotein-Related Kidney Disease: Glomerular Diseases Associated with Paraproteinemias. Clin. J. Am. Soc. Nephrol..

[32] El Ters M., Bobart S.A., Cornell L.D., Leung N., Bentall A., Sethi S., Fidler M., Grande J., Hernandez L.H., Cosio F.G. (2020). Recurrence of DNAJB9-Positive Fibrillary Glomerulonephritis After Kidney Transplantation: A Case Series. Am. J. kidney Dis..

[33] Dasari S., Alexander M.P., Vrana J.A., Theis J.D., Mills J.R., Negron V., Sethi S., Dispenzieri A., Highsmith W.E., Nasr S.H. (2018). DnaJ heat shock protein family B member 9 is a Novel biomarker for fibrillary GN. J. Am. Soc. Nephrol..

